# Male and undernourished children were at high risk of anemia in Ethiopia: a systematic review and meta-analysis

**DOI:** 10.1186/s13052-018-0513-x

**Published:** 2018-07-11

**Authors:** Mulugeta Melku, Wubet Worku Takele, Degefaye Zelalem Anlay, Daniale Tekelia Ekubagewargies, Zegeye Getaneh, Molla Abebe, Zegeye Abebe

**Affiliations:** 1 Department of Hematology and Immunohematology, School of Biomedical and Laboratory Sciences, College of Medicine and Health Sciences, University of Gondar, P.O. Box 196, Gondar, Ethiopia; 2 School of Nursing, College of Medicine and Health Sciences, University of Gondar, Gondar, Ethiopia; 3 Department of Clinical Chemistry, School of Biomedical and Laboratory Sciences, College of Medicine and Health Sciences, University of Gondar, P.O. Box 196, Gondar, Ethiopia; 4 Department of Human Nutrition, Institute of Public Health, College of Medicine and Health Sciences, University of Gondar, P.O. Box 196, Gondar, Ethiopia

**Keywords:** Anemia, Children, Ethiopia, Meta-analysis

## Abstract

**Background:**

Anemia is one of the global public health problems affecting more than one-third of the world population. It has been strongly associated with limited psychomotor development; and poor growth and performance in cognitive, social, and emotional function in children. Despite published data revealed that anemia is a public health problem among children in Ethiopia, there is no a pooled national estimate on the prevalence and associated risk factors of anemia.

**Methods:**

Published articles until December 31, 2017, were searched using comprehensive search strings through PubMed/Medline, EMBASE, SCOPUS, HINARI, Web of Science, Google Scholar and Google. Reference probing of published articles and hand searching were employed for grey literature. Two groups of review authors independently appraised the studies for eligibility and extracted the data. The quality of articles was assessed using Joana Brigg’s institute critical appraisal checklist for prevalence and analytical studies. The pooled estimates were determined using random effect model. Heterogeneity between the included studies was assessed using the I^2^ statistics. Subgroup analysis was employed in the evidence of heterogeneity. Publication bias was assessed by visual inspection of the funnel plot and Egger’s regression test statistic.

**Results:**

Of the total 871 articles retrieved, 34 articles which involved 61,748 children were eligible for meta-analysis. The overall pooled prevalence of anemia using random effect model was 31.14% (95% CI: 24.62, 37.66%). In subgroup analysis, the pooled prevalence of anemia was higher among preschool-aged children (44.17%; 95% CI: 37.19, 51.15%) than school-aged children (22.19%; 95% CI: 17.54, 26.83%). Furthermore, the odds of anemia was higher among children who were male (OR = 1.11; 95% CI: 1.03, 1.19), stunted (OR = 1.95; 95% CI: 1.52, 2.51), and wasted (OR = 2.05; 95% CI: 1.36, 3.10).

**Conclusion:**

The pooled prevalence of anemia among children was high, indicating that it had been continuing to be a public health problem. Therefore, there is a need to design a comprehensive prevention and control strategies to reduce its burden.

**Electronic supplementary material:**

The online version of this article (10.1186/s13052-018-0513-x) contains supplementary material, which is available to authorized users.

## Background

Anemia is a pathological condition in which the number and size of red blood cells or the hemoglobin concentration of red blood cells drops below an established cutoff value. Anemia can be caused by Iron, Folate, vitamin B12 and vitamin A deficiency, chronic inflammation, parasitic infections, and inherited disorders [1–3]. In developing countries, anemia can also be resulted from a number of causes; but nutritional deficiency particularly iron deficiency is the most common cause.

According to the World Health Organization (WHO) report, anemia is considered to be an indicator of both poor nutrition and health [1]. It is recognized as a major public health problem globally, mostly affecting children, women of childbearing age and pregnancy [4]. The health implication of anemia is numerous. It has a negative impact on mental and physical development, coordination, language development, and scholastic achievement [5–7]. It reduces the immunity which leads to susceptibility to infectious diseases and causes premature death. In addition, the consequences of anemia can be considered from a variety of perspectives, including the detrimental impact on economic and social development [8]. Compared to adults, the effect of anemia on children is horrible as their body is developing. Anemia is the most often hidden deficiency, with a few overt symptoms [1–3, 9]. Inspite of this, policymakers and health service providers often fail to recognize the massive economic costs and health consequences.

The prevalence of anemia remains high and is of priority area in low-income countries. According to WHO 2015 report, about 43% of under-five children were anemic, with regional variations of 62.3% in African, 53.8% in South-East Asia and 21.9% in Western Pacific Region [1].

According to the global estimates 2011, the prevalence of anemia among infants and children aged 6–59 months was 40–59.9% in Ethiopia [1]. Similarly, studies conducted in different parts of Ethiopia indicate that the overall prevalence of anemia ranges 10.7–43.7% [2, 3, 10–12] among school-age children.

Even though the prevalence of severe to moderate anemia in the last fifteen years has significantly declined in Ethiopia, children and pregnant women are still suffering from the consequences of anemia due to high iron requirements, low intake of iron from foods, and frequent episodes of infection [12, 13]. As many as six in ten under-five children in Ethiopia are anemic. But according to the local conditions, the proportion varies from region to region in the country. For example, the highest level of childhood anemia was found in Somali Region (83%), followed by Afar (75%) and Dire Dawa (72%), but the lowest was found in the Amhara Region (42%) [14].

The government of Ethiopia has been working to end childhood anemia. Accordingly, it endorsed national nutrition and bimanual school deworming programs; developed micronutrient deficiency prevention and control guideline. But studies from different corners of the country show that childhood anemia is still a major public health problem in Ethiopia. In addition, there is no a single national figure about childhood anemia and also inconclusive evidence about factors associated with it. Systematic review and meta-analysis generates concrete evidence in which it may urge policymakers and program managers to design an appropriate intervention to control and minimize the negative consequence of anemia. There is no meta-analysis conducted showing the pooled prevalence of childhood anemia in Ethiopia so far. Therefore, the aim of this review is to estimate the pooled prevalence and identify factors associated with anemia among children in Ethiopia.

## Methods

### Settings

This systematic review and meta-analysis was conducted in Ethiopian setting. Ethiopia is one of the developing countries located in the horn of Africa having nearly 100 million people with an area of 1,100,000 km^2^, making it the 27th largest country in the world. The country is working in reduction of maternal and child under-nutrition through implementing different programs and working jointly with international partners.

### Design and protocol registration

This systematic review was designed according to the Preferred Reporting Items for Systematic Reviews and Meta-Analysis Protocols (PRISMA-P 2015 Guidelines) [15]. The protocol has been registered in the PROSPERO, with the registration number of CRD42018088223.

### Search strategy

Relevant articles published until December 31, 2017 were searched in PubMed/Medline, HINARI, SCOPUS, EMBASE and Web of Sciences electronic databases. Grey literature were searched in Google Scholar and Google. The search terms were developed in accordance with the Medical Subject Headings thesaurus using the following terms in combination with free text key terms, “anemia”, “iron deficiency anemia”, “nutritional anemia”, “hemoglobin”, “nutritional status”, “hematological parameters” “children”, “preschool” “adolescent”, “determinant factors of anemia”, “associated factors of anemia”, and “Ethiopia”. The above terms were searched by a combination of Boolean operators (AND, OR). Hand searching of articles published in Ethiopian journal of health sciences, Ethiopian Medical Journal, Ethiopian Journal of Health and Development, and Ethiopian Journal of Health and Biomedical Sciences was conducted. Reference lists of retrieved articles were probed to identify any studies that are not retrieved from electronic databases.

### Study selection and quality appraisal

All articles retrieved through search strategy were imported to EndNote X7 (Thomson Reuters, New York, USA). After excluding duplicated articles, titles/abstracts were independently screened by two groups of review authors: group one (MM, ZA) and group two (DTE, WWT). Differences were resolved through thorough discussion. In case of disagreement between the two groups of review authors, the decision was determined by the third group of review authors (ZG, MA, DZA). For articles deemed to appear relevant during title/abstract screening, the full-text was appraised for inclusion in systematic review and meta-analysis. The quality of articles was assessed using Joana Brigg’s institute (JBI) critical appraisal tools for simple prevalence [16] and analytical cross-sectional studies [17] having nine and eight checklist items, respectively. The discrepancies during critical appraisal were solved as a similar manner for title/abstract screening phase.

### Outcomes of the study

The primary outcome of this review was to estimate the pooled prevalence of anemia among children in Ethiopia. The prevalence was calculated by dividing the number of anemic cases for the total number of children. Also, three determinant factors including sex, stunting, and wasting were assessed. Accordingly, the odds of developing anemia among male, stunted, and wasted children were calculated.

### Eligibility criteria

#### Inclusion criteria were:

- Studies published until December 31, 2017
- Studies reported the outcome of interest among children in Ethiopia
- Observational studies like cross-sectional, prospective cohort studies and repeated cross-sectional studies

#### Exclusion criteria were:

- Studies conducted in healthcare facilities
- Studies that used visual comparative method (Sahli-hellige method, and MBS hemoglobinometer color scale) and Copper Sulphate densitometer to ascertain the outcome (anemia)
- Studies conducted among children living with HIV/AIDS

### Data extraction

The JBI tool was adopted for data extraction. Information such as the name of first author, year of publication, age group of children, year of study, study area/region, study design, the total number of children, number of anemic case, and number of anemic and non-anemic cases for the reported associated factors were extracted. The data were recorded in Microsoft Excel spreadsheet. When authors found multiple publications from the same dataset, the article that reported the prevalence and associated factors in an extractable form were included. Moreover, for prospective cohort and repeated cross-sectional studies, the baseline data were used for our systematic review and meta-analysis to facilitate comparability of results across studies and to reduce a loss to follow-up bias.

### Data analysis

The data were entered into Microsoft Excel, and then exported to STATA version 14 (Stata Corp LLC, Texas, USA) for analysis. The magnitude of heterogeneity between included studies was quantitatively measured by the index of heterogeneity (I^2^ statistics) [18]. The I^2^ value of 25, 50 and 75% are assumed to represent low, medium and high heterogeneity, respectively. The significance of heterogeneity was determined by a *p*-value of I^2^ statistics, and a *p*-value < 0.05 was considered as an evidence of heterogeneity. When the I^2^ value was greater than 50%, we used Dersimonian and Liard random effect model to determine the pooled estimate [19]. Subgroup analyses were done considering age group and region as a grouping variables. Publication bias was evaluated using the visual funnel plot, and Egger’s statistics in the random effect model. Odds ratio with its 95% confidence was used to estimate the measure of association between anemia and associated factors. The results were presented in text and Forest plot.

## Results

### Description of studies

**Fig. 1:**
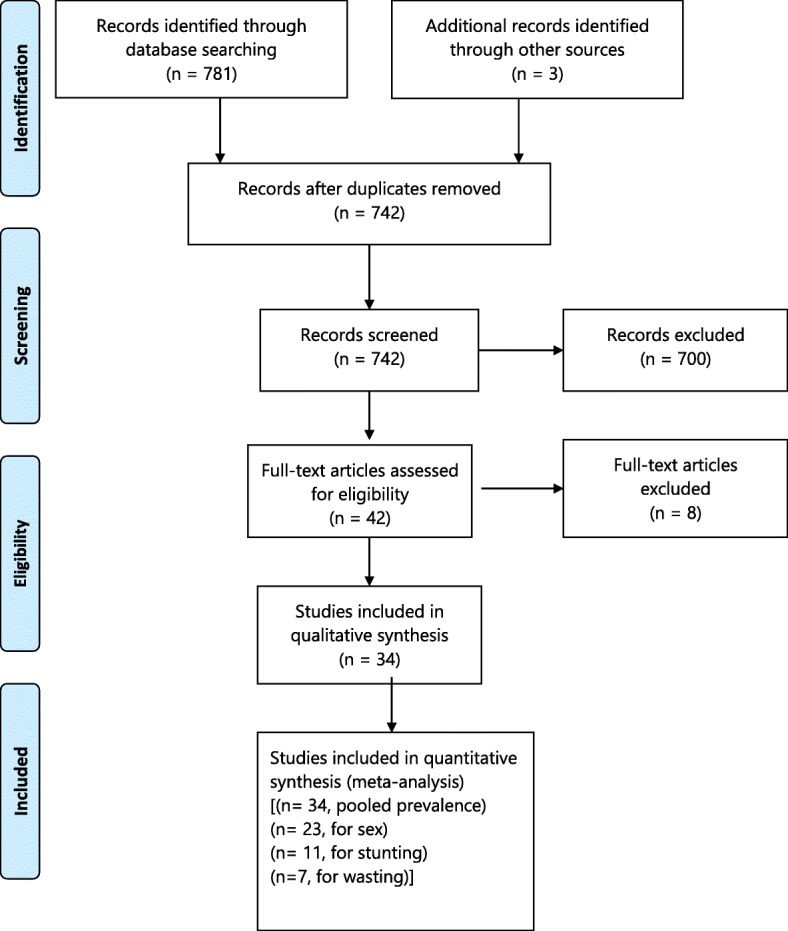
PRISMA flow diagram indicating the study selection for inclusion in the systematic review

A total of 781 records were retrieved, of which 38 articles were removed due to duplication. Seven hundred articles were also removed as their titles and abstract was irrelevant to the current analysis. The remaining 42 full-text articles were assessed for eligibility based on the pre-set criteria (Fig. 1).

Out of 42 studies, 8 were excluded: 4 studies were duplicated publications [20–23]; 2 studies were difficult to extract the outcome variables [24, 25]; and 2 studies were conducted in healthcare settings [26, 27].

In this review, 29 published studies, 2 demographic and health survey and 3 grey literature, which involved a total of 61,748 study participants, were included. Thirty-three (97.1%) and 1(2.9%) of the included studies were cross-sectional, and repeated cross-sectional studies, respectively. Concerning study settings where the studies were conducted, it has been as follows: 26.5% in Amhara Regional State ([11, 12, 28–32] Engidaye G, Melku M, Yalew A, Getaneh Z, Asrie F, Enawgaw B: Prevalence of anemia and associated factors among preschool-aged-children in Menz Gera Midir district, eastern Amhara, Ethiopia: a community based cross sectional study, submitted; Melku M, Alene KA, Terefe B, Enawgaw B, Melak T, Biadgo B, et al: Anemia Severity among Children Aged 6–59 Months in Gondar Town, Ethiopia: a community-based cross-sectional study, submitted); 17.6% in South Nations and Nationalities Regional State ([33–37]; Chane H: Magnitude of anemia and its contributing factor among school age children in Mihur Aklil district, Gurage zone. Ethiopia, unpublished]) and 20.6% in Oromia Regional State [2, 3, 13, 38–42]; 8.8% in Tigray Regional State [43–45]; 17.6% were national studies [14, 40, 46–49]; and 5.9% in Somali Regional State [10, 50]. In addition, 2.9% studies were conducted in Addis Ababa [51]. Of all included studies, about 59.4 and 37.5% of the studies were conducted among school-aged and preschool-aged children, respectively.

Regarding the association between anemia and sex, 23 studies were included; of which, 76.2% of the studies exhibited that there is no association between anemia and the sex of the children. Similarly, 11 studies were included to estimate the association between anemia and stunting. Around 82% of the studies revealed that the odds of anemia was higher among stunted children compared to non-stunted children. Furthermore, 5 studies reported that the likelihood of being anemic was higher among wasted children compared to their counterparts.

### Pooled prevalence of anemia

**Fig. 2:**
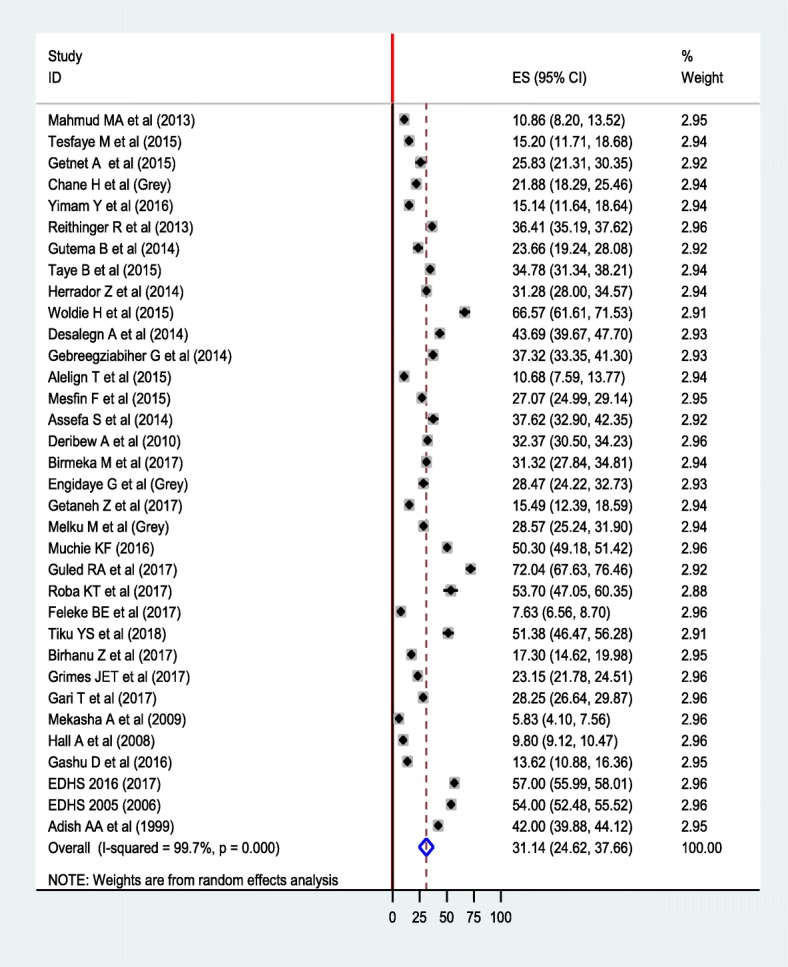
Forest plot of the pooled prevalence of anemia among children using random effect model

**Fig. 3:**
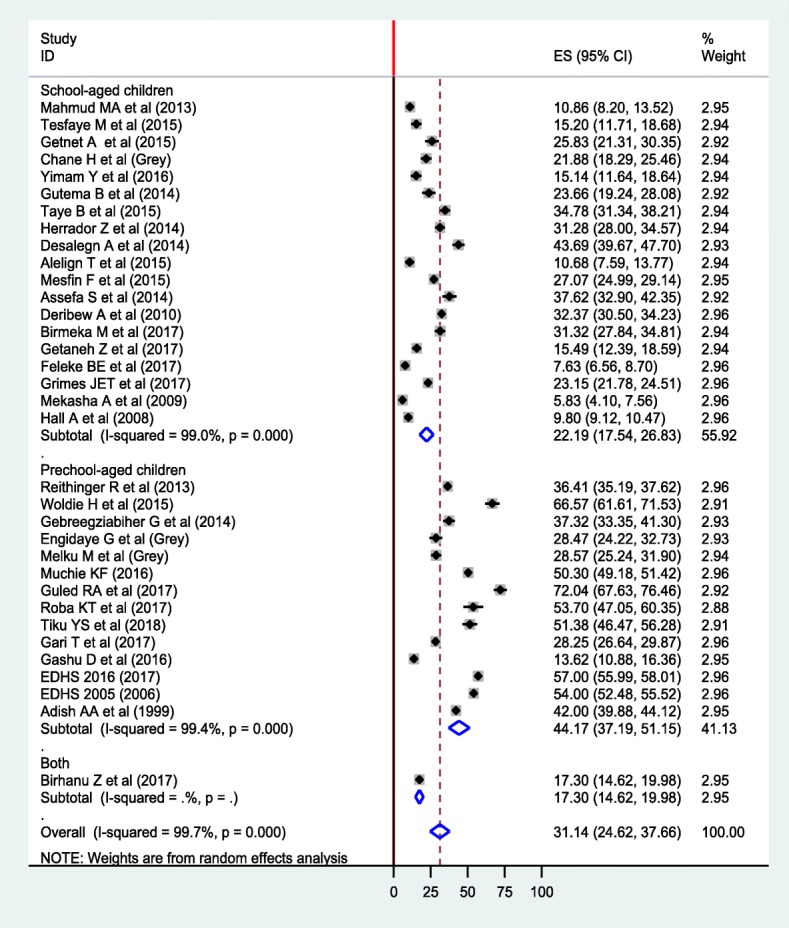
Forest plot of subgroup analysis of anemia among preschool-aged and school-aged children using random effect model

**Fig. 4:**
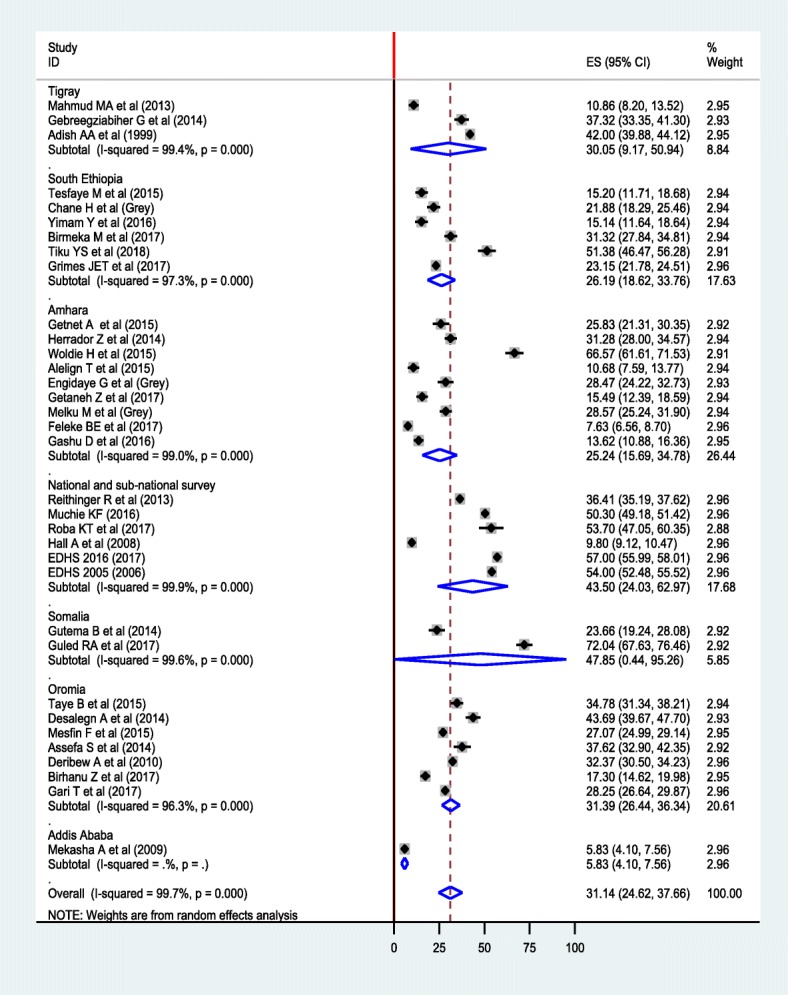
Forest plot of subgroup analysis of anemia by region using random effect model

The overall pooled prevalence of anemia using random effect model was 31.14% (95% CI: 24.62, 37.66%) (Fig. 2). We used a Funnel plot as well as Egger’s test of the intercept to check publication bias. The funnel plot is symmetrical (Additional file 1: Figure S1); and the Egger’s test result was 23.29 (95% CI: 11.32, 35.26, *p* = 0.387) (Additional file 2: Figure S2), indicating that there is no publication bias. We also assessed heterogeneity of the reported studies using the I^2^ statistics and it was significant (I^2^ = 99.7%, *p* < 0.001), showing a high level of heterogeneity between the included studies. Subgroup analyses were done by considering age and region as grouping variables. Accordingly, the pooled prevalence of anemia among preschool-aged children was 44.17% (95% CI: 37.19, 51.15%), whereas it 22.19% (95% CI: 17.54, 26.83%) among school-aged children (Fig. 3). In terms of region, the highest and the lowest prevalence was found in Somalia region (47.85%) and Addis Ababa (5.83%), respectively (Fig. 4). In addition, a sensitivity analysis was done and the result showed that there was no a single estimate out of the combined estimate’s 95% confidence interval/range.

### Association between anemia, sex, and undernutrition

**Fig. 5:**
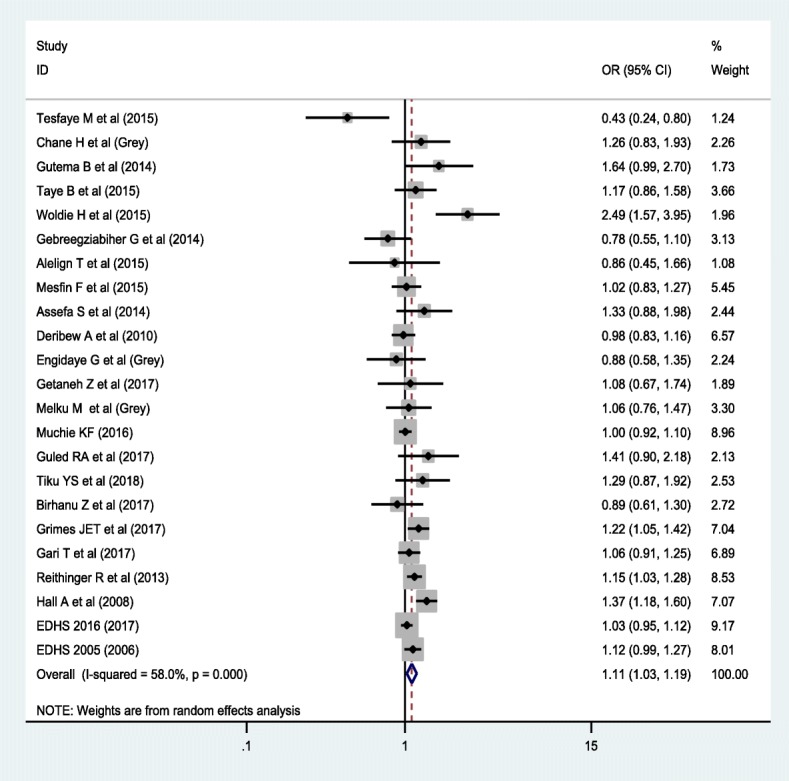
Forest plot of the association between anemia and male sex using random effect model

In random effect model, the pooled effect size of anemia among males was 1.11 (OR = 1.11; 95% CI: 1.03, 1.19) times higher compared to females (Fig. 5).

**Fig. 6:**
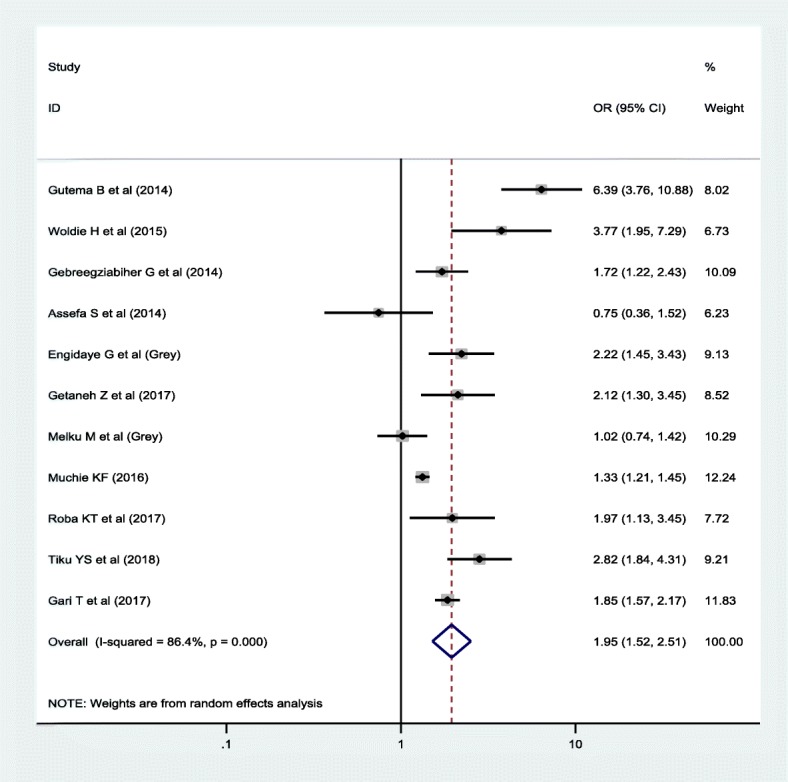
Forest plot of the association between anemia and stunting using random effect model

**Fig. 7:**
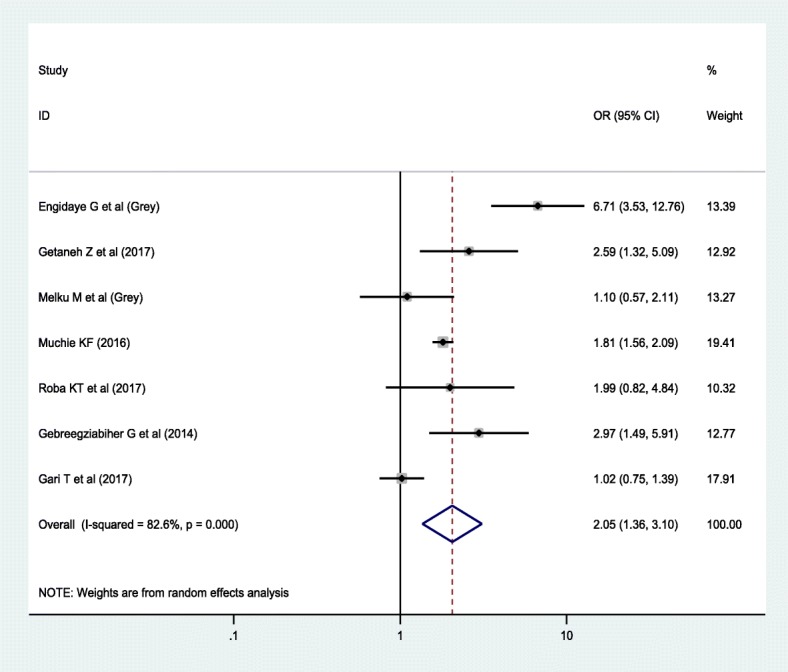
Forest plot of the association between anemia and wasting using random effect model

The result showed that nutritional status has a significant association with anemia. Accordingly, higher odds of being anemic was found among stunted children compared to their counterparts (OR = 1.95; 95% CI: 1.52, 2.51) (Fig. 6). On the other hand, wasted children were 2.05 times (OR = 2.05; 95% CI: 1.36, 3.10) more likely to be anemic compared to non-wasted children (Fig. 7).

## Discussion

Anemia has been a public health problem affecting the low, middle and high-income countries. It has also been significantly associated with negative consequences on health, social and economic development [52, 53]. Globally, it is a moderate to a severe public health problem in children. As to the WHO 2015 estimate, the highest prevalence of anemia is found in children, 42.6% (95% CI: 37, 47%) [54]. The consequence of anemia is worse in children as it limits the physical growth [55], metal [56], social [57] as well as the behavioral development [58].

The overall pooled prevalence of anemia was 31.14% (95% CI: 24.62, 37.66%). This implies that anemia is a moderate public health problem among children in Ethiopia [59], which needs to design intervention and control strategies in a comprehensive approach to reduce its burden. The estimated prevalence is consistent with the global age-standardized prevalence of anemia in 2013 (27.0%) and in 1990 (33.3%) [60], and global age-unstandardized prevalence in 2010 (32.9%) [61].

In subgroup analysis, the pooled prevalence of anemia among school-aged children was 22.19% (95% CI: 17.54, 26.83%), which is lower than pooled estimate among preschool-aged children, 44.17% (95% CI: 37.19, 51.15%). It is reasonable that the magnitude of anemia declines as the age of children advanced. Compared to school-aged children, preschool-aged children have high dietary requirement owing to rapid rate of growth [39] and expansion of blood volume, which lead to nutritional imbalance. Moreover, the biannual school-based deworming program that the government of Ethiopia recently implemented, and water, sanitation, and hygiene (WASH) program are substantially contributing for low parasitic infestation [37, 59, 62], and ultimately reduced the prevalence of anemia among school-aged children.

In random effect model, the pooled effect size of anemia among male children is higher than female children (OR = 1.11; 95% CI: 1.03. 1.19). Consistent with previous studies [63, 64], the risk of anemia is high in male than female children. Growth and gonadal hormones are the key role players of growth and development in children, and pre-pubertal and pubertal boys and girls [65]. Despite lower than adults, evidence suggested that the level of testosterone is higher in boys than girls during pre-puberty stage [66], and in early pubertal and pubertal development [67]. Testosterone is a known stimulator of erythropoiesis and enhanced metabolism [68]. High level of testosterone in boys stimulates growth velocity so that boys have high nutritional requirement than girls. Concurrently, undernutrition is a common problem among children in Sub-Saharan countries including Ethiopia [11, 69–71] which may lead to the development of nutritional deficiency anemia, particularly iron deficiency anemia, in boys than girls.

In this meta-analysis, a higher odds of anemia was noted among stunted (OR = 1.95; 95% CI: 1.36, 2.51) and wasted (OR = 2.05; 95% CI: 1.36, 3.10) children. Previous literature also supported that stunting [12, 72–74] and wasting [43] are a strong predictors of childhood anemia. In the developing nations, where a diverse supply of foods is limited, macro and micronutrients deficiencies are the common public health problems [75]. As the result, stunting and wasting, which are the indicator of chronic and acute malnutrition, respectively, have imposed a great challenge on child health [76]. One of which is anemia that can be resulted from inadequate intake of both macro and micronutrients such as iron, B12 and folate, which are important for the formation of blood cells [77]. Besides, undernutrition impairs the immunity in which children become susceptible to infectious diseases. The situation may cause a loss of nutrients, malabsorption, underutilization of bioavailable nutrients, blood loss and immune-mediated destruction of RBCs, which have been associated with low level of hemoglobin [78].

The review has some limitations. The extent of heterogeneity between included studies was high, which can be attributed to differences in methodology, study period, and geographic location. Moreover, some of the studies did not consider an altitudinal adjustment to define anemia, which may underestimate the burden of anemia among children. Given these limitations, the review was conducted according to the preferred reporting items for systematic review and meta-analysis (PRISMA-P statement) protocol. Besides, a comprehensive searching with no language restriction and the involvement of experts from biomedical, public health and clinical fields improved quality of evidence generated.

## Conclusion

In conclusion, the pooled national prevalence of anemia among children was high. As to the WHO classification of anemia on the basis of public health importance, it is a moderate public health problem among children in Ethiopia. The pooled prevalence was higher among preschool-aged children than school-aged children. Moreover, the likelihood of anemia was higher among male and undernourished children. Therefore, optimizing the nutritional supplementation, improving access to child health care, and sustaining the socio-economic development need to be emphasized to reduce the burden of childhood anemia in Ethiopia.

## Additional files

Additional file 1: **Figure S1.** Funnel Plot. The Funnel plot showing the pooled estimate of anemia among children in Ethiopia. This is a visual method to see small-study effect or publication bias. (DOC 26 kb)

Additional file 2: **Figure S2.** Egger’s Plot. A Plot of Egger’s test of publication bias for pooled estimate of anemia among children in Ethiopia. Egger’s test is a statistical method to test the presence of small-study effect or publication bias on the pooled estimate. (DOC 26 kb)
