# Supplementary material for: Male and undernourished children were at high risk of anemia in Ethiopia: a systematic review and meta-analysis
Source: Ital J Pediatr. 2018 Jul 11;44:79. doi: 10.1186/s13052-018-0513-x (PMC6042228; doi:10.1186/s13052-018-0513-x)
Supplement: Supplementary file 1 — Figure S1. Funnel Plot. The Funnel plot showing the pooled estimate of anemia among children in Ethiopia. This is a visual method to see small-study effect or publication bias. (DOC 26 kb) [file 13052_2018_513_MOESM1_ESM.doc]

**Additional figure file 1**: The Funnel plot showing the pooled estimate of anemia among children in Ethiopia
