# Supplementary material for: Male and undernourished children were at high risk of anemia in Ethiopia: a systematic review and meta-analysis
Source: Ital J Pediatr. 2018 Jul 11;44:79. doi: 10.1186/s13052-018-0513-x (PMC6042228; doi:10.1186/s13052-018-0513-x)
Supplement: Supplementary file 2 — Figure S2. Egger’s Plot. A Plot of Egger’s test of publication bias for pooled estimate of anemia among children in Ethiopia. Egger’s test is a statistical method to test the presence of small-study effect or publication bias on the pooled estimate. (DOC 26 kb) [file 13052_2018_513_MOESM2_ESM.doc]

**Additional figure file 2**: A Plot of Egger’s test of publication bias for pooled estimate of anemia among children in Ethiopia
